# Niemann–Pick C1-Like 1 in Cholesterol Absorption and Homeostasis: Mechanisms, Regulation, and Emerging Phytochemical Inhibitors

**DOI:** 10.3390/cimb48060592

**Published:** 2026-06-03

**Authors:** Pei-Yi Chen, Je-Wen Liou, Ming-Jiuan Wu, Jui-Hung Yen

**Affiliations:** 1 Laboratory of Medical Genetics, Genetic Counseling Center, Hualien Tzu Chi Hospital, Buddhist Tzu Chi Medical Foundation, Hualien 970374, Taiwan; pyc571@gmail.com; 2 Department of Molecular Biology and Human Genetics, Tzu Chi University, Hualien 970374, Taiwan; 3 Department of Biochemistry, School of Medicine, Tzu Chi University, Hualien 970374, Taiwan; jwliou@gms.tcu.edu.tw; 4 Department of Pharmacy, Chia Nan University of Pharmacy and Science, Tainan 717301, Taiwan; imwu@gm.cnu.edu.tw; 5 Institute of Medical Sciences, Tzu Chi University, Hualien 970374, Taiwan

**Keywords:** cholesterol homeostasis, ASCVD, MAFLD, cholesterol absorption, NPC1L1, phytochemicals

## Abstract

Disruption of cholesterol homeostasis is closely associated with hypercholesterolemia, dyslipidemia, atherosclerotic cardiovascular disease (ASCVD), and metabolic disorders such as metabolic dysfunction-associated fatty liver disease (MAFLD). Intestinal and hepatic cholesterol absorption are central to maintaining systemic cholesterol balance, with Niemann–Pick C1-Like 1 (NPC1L1) acting as a key transporter that mediates cholesterol uptake in enterocytes and hepatocytes. Aberrant NPC1L1 expression or activity promotes excessive cholesterol accumulation in both plasma and liver, thereby contributing to dyslipidemia and hepatic steatosis. Consequently, NPC1L1 has emerged as an important therapeutic target for reducing cholesterol absorption and improving lipid homeostasis. Although ezetimibe is currently the only clinically approved NPC1L1 inhibitor, its limited efficacy as monotherapy highlights the need for alternative or complementary therapeutic strategies. Growing evidence indicates that natural phytochemicals, particularly polyphenols and flavonoids, can modulate NPC1L1 at both transcriptional and functional levels. These compounds not only suppress intestinal cholesterol absorption but also attenuate hepatic lipid accumulation, ultimately improving circulating lipid profiles. This review summarizes recent advances in understanding the role of NPC1L1 in cholesterol metabolism and highlights the emerging therapeutic potential of phytochemicals as novel complementary approaches for the prevention and treatment of lipid metabolic disorders.

## 1. Introduction

Cholesterol is an essential structural component of cell membranes and serves as a precursor for the synthesis of steroid hormones, fat-soluble vitamins, and bile acids. These cholesterol-derived molecules are critical for diverse biochemical and physiological functions. Cholesterol homeostasis is tightly regulated through multiple biological processes, including hepatic biosynthesis, fecal excretion, bile acid production, and apolipoprotein B100 metabolism [1]. Precise regulation of cholesterol absorption and metabolism is therefore crucial for maintaining lipid balance. Disruption of this homeostasis leads to dyslipidemia and contributes to the development of diseases such as cancer and neurodegenerative disorders [2].

The coordinated regulation of cholesterol absorption by the intestine and liver plays a central role in maintaining systemic lipid homeostasis [3,4]. Excessive cholesterol absorption is strongly associated with metabolic disorders, including hypercholesterolemia, atherosclerotic cardiovascular diseases (ASCVD), and metabolic dysfunction-associated fatty liver disease (MAFLD) [5,6]. Mechanistic studies have identified Niemann–Pick C1-like 1 (NPC1L1) as a key transporter mediating cholesterol uptake in both enterocytes and hepatocytes. As such, NPC1L1 represents an important therapeutic target for managing dyslipidemia and reducing cardiovascular risk [7]. Ezetimibe, the only clinically approved NPC1L1 inhibitor, reduces intestinal cholesterol absorption by targeting NPC1L1, leading to an approximate 20% decrease in circulating low-density lipoprotein cholesterol (LDL-C). When combined with statin therapy, it further provides an absolute risk reduction of about 2% in major cardiovascular events among patients with acute coronary syndrome [8]. Collectively, NPC1L1 plays a pivotal role in whole-body cholesterol homeostasis and serves as a promising molecular target for the prevention and treatment of hypercholesterolemia, ASCVD, and MAFLD.

In this review, we summarize current advances in understanding the molecular functions of NPC1L1 in cholesterol homeostasis and its roles in dyslipidemia and hepatic steatosis. We further discuss the development of NPC1L1 inhibitors, with particular emphasis on natural phytochemicals that suppress NPC1L1-mediated cholesterol uptake by modulating its expression or activity, thereby regulating lipid metabolism and showing therapeutic potential for the prevention and treatment of lipid metabolic disorders.

## 2. The Importance of Cholesterol Absorption and Homeostasis

The gut and liver are the primary organs governing cholesterol absorption and metabolism [9]. A key mechanism in this process is the enterohepatic circulation, which facilitates the absorption and recycling of endogenous cholesterol. This process is primarily mediated by enterocytes, which take up cholesterol from the small intestinal lumen. Luminal cholesterol is derived from two main sources: dietary intake and biliary secretion [10]. Bile is composed mainly of bile salts, cholesterol, phospholipids, and bile pigments. These components are synthesized in hepatocytes, concentrated, and stored in the gallbladder. Upon release, bile delivers a significant amount of cholesterol into the intestinal lumen [11].

### 2.1. The Cholesterol Absorption and Homeostasis in Enterocytes

Intestinal cholesterol absorption by enterocytes positively correlates with plasma cholesterol levels. Several factors, including dietary, biliary, pharmacological, cellular, and luminal factors, may affect intestinal cholesterol absorption efficiency [12,13]. Recent studies have shown that cellular cholesterol homeostasis is regulated by cholesterol absorption and endogenous cholesterol synthesis, thereby maintaining the LDL-C/HDL-C ratio [14,15]. A human study found that cholesterol synthesis inhibitors reduced endogenous cholesterol synthesis while increasing cholesterol absorption [16]. These findings show that cholesterol absorption in the small intestine influences cholesterol homeostasis.

**Figure 1:**
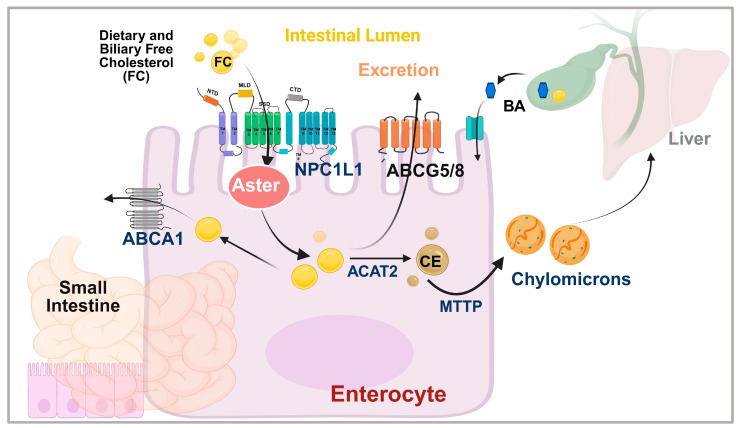
The cholesterol absorption and homeostasis in enterocytes. In the intestinal lumen, free cholesterol (FC) is solubilized, enabling it to approach the brush border membrane, where it is taken up into enterocytes via the NPC1L1 transporter. The absorbed cholesterol is then esterified by ACAT2 to form CE, which are subsequently assembled into chylomicrons with the assistance of MTTP and released into the lymphatic system. Excess FC can be effluxed back into the intestinal lumen by ABCG5/8. In addition, cholesterol can be exported from enterocytes via ABCA1, contributing to the formation of HDL. Created in BioRender. Liou, J.-W. (2026) https://BioRender.com/bucejkb (accessed on 27 April 2026).

Intestinal cholesterol absorption is a complex process involving multiple pathways and transport proteins. Figure 1 illustrates the process of cholesterol absorption in enterocytes. In the intestinal lumen, free cholesterol (FC) is first solubilized into bile salt micelles, which are complexes composed of bile acids (BA) and phospholipids. These micelles deliver cholesterol to the brush border membrane of enterocytes, where it is internalized via the NPC1L1 transporter [17]. During NPC1L1-mediated endocytosis, cholesterol accumulation at the plasma membrane leads to a localized increase in its concentration, which recruits Aster proteins (Aster-B and Aster-C) to the membrane surface. Once inside the enterocyte, cholesterol is transported via a non-vesicular pathway mediated by these proteins from the plasma membrane to the endoplasmic reticulum (ER) [18]. There, it is esterified by acyl-CoA: cholesterol acyltransferase 2 (ACAT2) to form cholesteryl esters (CE), which are subsequently incorporated into chylomicrons by microsomal triglyceride transfer protein (MTTP). These chylomicrons are then secreted into the circulation via the lymphatic system [19,20]. In the circulation, chylomicrons release fatty acids and are metabolized into chylomicron remnants, which are taken up by hepatocytes via specific receptors [21].

In intestinal epithelial cells, excess cholesterol can be directly excreted into the intestinal lumen. Specifically, surplus FC is effluxed across the apical membrane by the heterodimeric ATP-binding cassette transporters ABCG5 and ABCG8 [22], and is ultimately eliminated from the body via feces. In addition, reverse cholesterol transport (RCT) begins with the formation of nascent high-density lipoprotein (HDL) particles by the enterocytes. These nascent HDL particles acquire cholesterol that is effluxed from cells via ABCA1, leading to the formation of mature HDL. The mature HDL subsequently transports cholesterol back to the liver for metabolism [23].

### 2.2. The Cholesterol Absorption and Homeostasis in Hepatocytes

**Figure 2:**
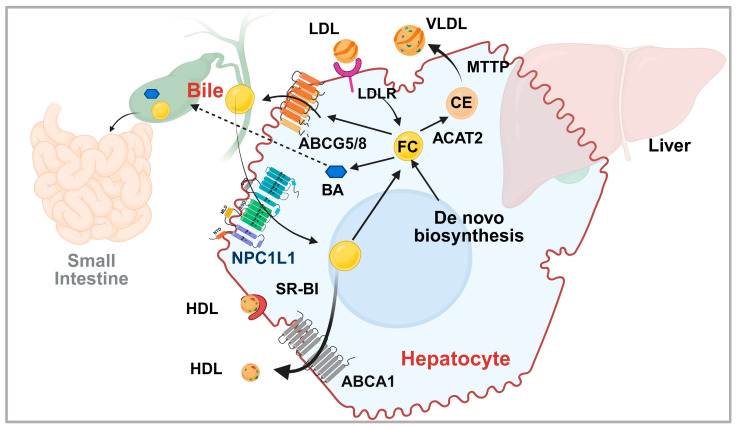
Hepatic cholesterol metabolism, transport, and biliary regulation. Hepatic cholesterol, derived from de novo synthesis or uptake of circulating lipoproteins, is either converted into bile acids for secretion or packaged into VLDL for release into the bloodstream. In circulation, LDL delivers cholesterol to tissues and is taken up by hepatocytes via LDL receptors (LDLR). In parallel, HDL mediates RCT, returning cholesterol to the liver through SR-B1-dependent uptake. Hepatic cholesterol is eliminated via bile through ABCG5/8-mediated efflux or conversion into bile acids, whereas NPC1L1 reabsorbs biliary cholesterol, thereby modulating cholesterol homeostasis. Created in BioRender. Liou, J.-W. (2026) https://BioRender.com/0ncoo22 (accessed on 28 April 2026).

In addition to intestinal absorption, hepatic cholesterol metabolism plays a crucial role in maintaining whole-body cholesterol homeostasis [24]. Figure 2 illustrates cholesterol metabolism in hepatocytes. Cholesterol synthesized de novo in hepatocytes or taken up from circulating lipoproteins contributes to the hepatic cholesterol pool. This cholesterol can be converted into bile acids and secreted into the intestine or packaged into very low-density lipoproteins (VLDL) for secretion into the bloodstream. In circulation, the triglycerides (TG) within VLDL particles are hydrolyzed by lipoprotein lipase (LPL) on the vascular endothelium, releasing free fatty acids (FFA) and converting VLDL into intermediate-density lipoproteins (IDL). IDL particles are further metabolized into LDL, which carries cholesterol in the blood. LDL cholesterol can be taken up by hepatocytes via LDL receptors (LDLR) for further metabolism [25]. Additionally, the HDL, produced by the intestine or other peripheral tissues, transports cholesterol back to the liver via the RCT pathway. This process involves direct interaction with the scavenger receptor class B type 1 (SR-B1) on the surface of hepatocytes, facilitating cholesterol uptake into liver cells, which are subsequently metabolized [23].

In the liver, free cholesterol can be directly secreted into bile or converted into more water-soluble bile acids for excretion. Hepatic ABCG5/8 promotes the efflux of cholesterol from hepatocytes into bile, thereby facilitating its elimination. In contrast, hepatic NPC1L1 functions to reabsorb biliary cholesterol. It is expressed on the canalicular (bile-facing) membrane of hepatocytes, where it mediates the uptake of cholesterol from bile back into hepatocytes, thus regulating biliary cholesterol levels. Consequently, hepatic NPC1L1 counteracts cholesterol elimination via bile, influences plasma cholesterol levels, and helps maintain cholesterol homeostasis [26,27].

## 3. Molecular Features of NPC1L1

NPC1L1 has been identified as the principal transporter responsible for cholesterol uptake in both enterocytes and hepatocytes. This highlights its central role in cholesterol homeostasis and underscores its therapeutic potential as a target for lipid-related disorders.

### 3.1. The Characteristics of NPC1L1

The *NPC1L1* gene was first identified using an integrated strategy combining genomic and bioinformatics approaches. Specifically, researchers performed a cross-analysis of expressed sequence tags (ESTs), transmembrane proteins, glycoproteins, and protein motifs potentially involved in cholesterol interaction to investigate cholesterol absorption [7]. The human *NPC1L1* gene spans approximately 29 kilobases and is located on chromosome 7p13. It undergoes alternative splicing to generate multiple transcript isoforms in addition to the full-length form. The major isoform lacks exon 15 and encodes a protein consisting of 1332 amino acids with a molecular weight of approximately 140 kDa. In contrast, other splice variants include an exon 15-containing isoform and a truncated isoform lacking exon 7 [11]. The NPC1L1 protein shares about 51% amino acid sequence similarity with Niemann–Pick C1 (NPC1), a protein associated with lipid storage disorders [28]. NPC1L1 is a highly N-glycosylated membrane protein containing 13 transmembrane domains and functions as a transporter involved in cholesterol uptake. Notably, species-specific differences in the tissue distribution of NPC1L1 expression have been reported. In humans, NPC1L1 is highly expressed in both the liver and intestine. In contrast, in rodents such as mice, NPC1L1 expression is predominantly restricted to the intestine, with minimal expression in the liver [11]. These differences suggest that hepatic NPC1L1 may play a distinct physiological role in humans.

### 3.2. Structural Features and Functions of NPC1L1

**Figure 3:**
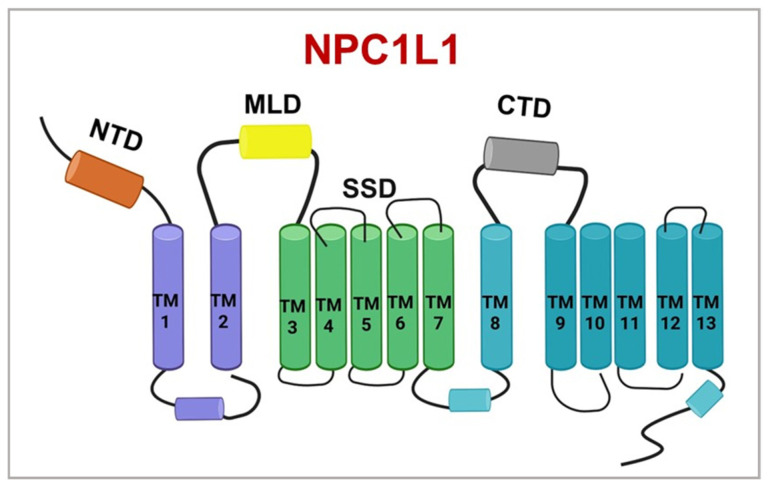
The protein structure of NPC1L1. The NPC1L1 protein contains the NTD, TMD (TM1-TM13), the MLD, the SSD and the CTD. Created in BioRender. Liou, J.-W. (2026) https://BioRender.com/0ncoo22 (accessed on 25 April 2026).

In enterocytes, NPC1L1 facilitates the absorption of dietary and biliary free cholesterol from the intestinal lumen, whereas in hepatocytes, it promotes cholesterol reabsorption from bile canaliculi, thereby reducing biliary excretion. These functions are essential for maintaining plasma and hepatic cholesterol homeostasis, as well as regulating LDL-C levels and overall lipid metabolism [11]. Early structural insights into NPC1L1 were derived from studies of the homologous NPC1 protein. Subsequent characterization of NPC1L1 has advanced understanding of the molecular mechanisms underlying NPC1L1-mediated cholesterol absorption [29]. NPC1L1 protein is topologically similar to the resistance-nodulation-division (RND) family of bacterial permeases, which mediate the efflux of lipophilic compounds like fatty acids and bile acids out of bacteria. This structural similarity supports a key role for NPC1L1 in lipid transport [30]. Recent cryo-electron microscopy (cryo-EM) studies have identified several key structural domains of NPC1L1, including the N-terminal domain (NTD), 13 transmembrane domains (TMDs), the sterol-sensing domain (SSD), the middle luminal domain (MLD), and the C-terminal domain (CTD) (Figure 3). The NTD contains a cholesterol-binding cavity, whereas the MLD serves as the binding site for the cholesterol absorption inhibitor ezetimibe [31].

### 3.3. The Mechanism of NPC1L1-Mediated Cholesterol Absorption

**Figure 4:**
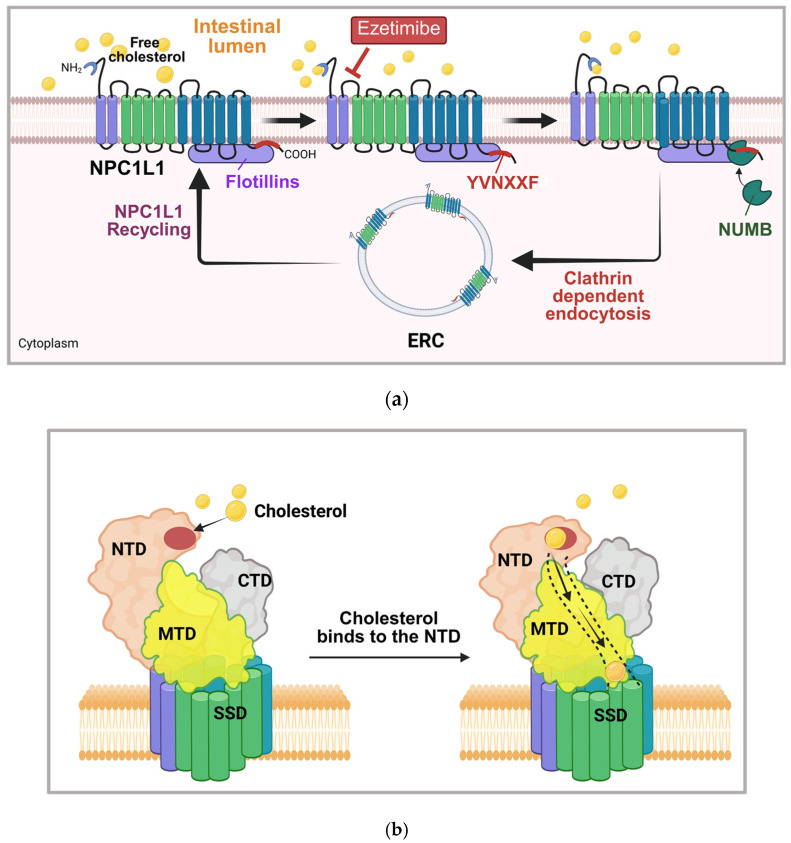
The hypothetical mechanism of NPC1L1-mediated cholesterol absorption. (**a**) The binding of free cholesterol to the NTD of NPC1L1 promotes the formation of microdomains. It subsequently triggers conformational changes in NPC1L1 so that the endocytic motif YVNXXF can be recognized by NUMB, which facilitates clathrin-mediated endocytosis. The endocytic vesicles migrate to the endocytic recycling compartment (ERC). Upon cholesterol dissociation, NPC1L1 can be recycled back to the plasma membrane. Ezetimibe binds to the second extracellular loop of NPC1L1 and inhibits intestinal cholesterol uptake. (**b**) NPC1L1-mediated cholesterol uptake via a non-endocytic pathway. Cholesterol binds to the NTD of NPC1L1, triggering a conformational change that opens an internal tunnel connecting to the SSD within the TMD. Created in BioRender. Liou, J.-W. (2026) https://BioRender.com/ah7pzru (accessed on 26 April 2026).

As illustrated in Figure 4a, cholesterol uptake is proposed to occur through binding to NPC1L1, followed by internalization via an endocytosis-dependent mechanism [32]. Accordingly, NPC1L1 localizes to both the plasma membrane and the intracellular endocytic recycling compartment (ERC), with its subcellular distribution regulated by cellular cholesterol levels. Under cholesterol-replete conditions, NPC1L1 undergoes endocytic recycling, whereas cholesterol depletion promotes its redistribution to the cell surface, thereby enhancing cholesterol uptake [33]. Cholesterol binding to the NTD of NPC1L1 induces conformational changes that promote the formation of membrane microdomains enriched in cholesterol, flotillins, and gangliosides. Upon cholesterol restoration, the NPC1L1-cholesterol complex is internalized from the plasma membrane to the ERC via a flotillin-dependent pathway [34]. In addition, gangliosides in the outer leaflet of the plasma membrane may facilitate the recruitment of NPC1L1 and flotillins, contributing to the formation of functional membrane microdomains essential for cholesterol transport [35]. This process triggers a conformational change that exposes an endocytic signaling motif (YVNXXF) in the C-terminal of NPC1L1. This motif is recognized by the clathrin adaptor protein NUMB, which subsequently mediates the clathrin/AP2-dependent endocytosis of the NPC1L1-cholesterol complex. Once inside the endosomes, cholesterol dissociates from NPC1L1. The released cholesterol can then activate GTP-bound Cdc42, while NPC1L1 interacts with myosin Vb through the LIM domain and actin-binding protein 1 (LIMA1). This specific interaction enables NPC1L1 to recycle from the ERC back to the plasma membrane, facilitating continuous cycles of cholesterol binding and uptake [34,36].

Figure 4b depicts an alternative, endocytosis-independent mechanism of cellular cholesterol uptake, whereby cholesterol binds to NPC1L1 and is transported into the cell through a dedicated channel. Current evidence suggests that TMD of NPC1L1 harbors a sterol-sensing domain (SSD), which is functionally connected to the NTD via an internal cholesterol transport tunnel. Cholesterol binding to the NTD may induce a conformational change that opens this tunnel, allowing cholesterol to be transferred to the SSD and subsequently inserted into the membrane. This mechanism provides a direct pathway for cholesterol to move from the NTD to the plasma membrane, where it can integrate into the lipid bilayer. This uptake process is dynamically regulated by intracellular cholesterol levels [37,38,39]. Additionally, recent studies suggest that hepatic NPC1L1 may interact with canalicular membrane proteins, such as caveolin-1, to protect the membrane from the detergent effects of bile salts and maintain cholesterol homeostasis [40].

Despite these advances, the structural and functional properties of NPC1L1 remain to be fully elucidated. These structural insights are critical for understanding cholesterol internalization and for the rational design of selective NPC1L1 inhibitors.

### 3.4. The Genetic Variants of NPC1L1 in Cholesterol Absorption

Several single nucleotide polymorphisms (SNPs) and other polymorphic variants in the NPC1L1 gene have been associated with cholesterol absorption efficiency, plasma total cholesterol and LDL-C levels, thereby influencing cholesterol homeostasis, susceptibility to dyslipidemia and coronary heart disease risk. Previous studies have shown that individuals with low intestinal cholesterol absorption are more likely to carry non-synonymous variants of the NPC1L1 gene compared with high absorbers [41]. Specifically, SNPs such as rs2072183, rs17655652, rs41279633, rs217434, and rs3187907 alter intestinal cholesterol absorption, plasma LDL-C concentrations, and therapeutic responses to lipid-lowering treatments [42,43,44].

## 4. Regulation of *NPC1L1* Gene Expression

In humans, the *NPC1L1* gene is primarily expressed in enterocytes and hepatocytes, where both shared and tissue-specific factors regulate its transcription. Several transcription factors have been identified to control *NPC1L1* promoter activity in these cell types [11]. Among them, sterol regulatory element-binding protein 2 (SREBP2) and hepatocyte nuclear factor-1α (HNF-1α) are the principal regulators in both the intestine and the liver [45,46]. In the human Huh-7 hepatoma cell line, both SREBP2 and HNF-1α act as key transcriptional activators of *NPC1L1* gene expression [46]. HNF-4α, an upstream regulator of HNF-1α, acts synergistically with SREBP2 to enhance *NPC1L1* expression in Caco-2 and HepG2 cells [47]. Consistently, reduced expression of HNF-4α and SREBP2 is associated with decreased intestinal NPC1L1 levels in mice fed a high-cholesterol diet (HCD) [48]. Similarly, liver receptor homolog-1 (LRH-1) binds to the *NPC1L1* promoter and cooperates with SREBP2 to further enhance its transcription [49].

In contrast, activation of liver X receptor (LXR) suppresses *NPC1L1* expression. Treatment with the LXR agonist T0901317 reduces *NPC1L1* expression and dietary cholesterol absorption in both intestinal cell models and mice [50]. Peroxisome proliferator-activated receptors, including PPARα and PPARδ also modulate NPC1L1, although their effects appear to be context-dependent [51,52]. Activation of PPARα or PPARδ, has been shown to reduce *NPC1L1* expression and intestinal cholesterol absorption. In fenofibrate-treated mice, cholesterol absorption decreased by 35–47%, accompanied by increased fecal sterol excretion and reduced *NPC1L1* expression in the proximal intestine [53,54]. These findings suggest that NPC1L1 is negatively regulated by LXR- and PPAR-dependent pathways. In addition, cyclic AMP-responsive element-binding protein H (CREBH) has been identified as a suppressor of *NPC1L1* expression in enterocytes, contributing to reduced cholesterol absorption and improved lipid homeostasis [55]. These findings demonstrate that NPC1L1 transcription is governed by a complex regulatory network and suggest that transcription factors regulating its expression may serve as promising therapeutic targets for cholesterol metabolism.

In addition to transcriptional and signaling pathway-mediated regulation, post-transcriptional control by microRNAs (miRNAs) has emerged as an important regulator in lipid metabolism and cholesterol homeostasis [56]. Several lipid metabolism-regulating miRNAs, including miR-33, miR-122, miR-27, and miR-223, have been implicated in the coordinated regulation of cholesterol transporters, cholesterol and fatty acid synthesis, and other lipid metabolic genes [57].

To date, no miRNAs directly targeting NPC1L1 have been identified; however, a subset of miRNAs may modulate its expression indirectly by regulating upstream transcription factors. miR-33a/b, located within SREBP introns, act in concert with these transcription factors to regulate cholesterol synthesis, uptake, and transport, thereby contributing to cellular cholesterol homeostasis [58]. Under lipid-induced metabolic stress, hepatic miR-34a represses the key transcription factor HNF-4α [59]. Meanwhile, miR-7, which is activated by the fatty acid regulator PPARα, promotes sterol biosynthesis by upregulating SREBP1 and SREBP2. Through this mechanism, miR-7 acts as a critical link between the PPAR, SREBP, and LXR signaling pathways in regulating hepatic cholesterol and fatty acid metabolism [60]. Collectively, these miRNAs may play a critical role in modulating NPC1L1 expression and overall cholesterol homeostasis, and may also serve as therapeutic targets for dyslipidemia.

## 5. Dysregulation of Lipid Metabolism Driven by NPC1L1

Aberrant NPC1L1-mediated cholesterol uptake in the intestine and liver contributes to hypercholesterolemia, atherosclerosis, and metabolic disorders such as hepatic steatosis, MAFLD, obesity, and diabetes [61]. Conversely, reduced cholesterol uptake has been observed in NPC1L1-knockout Caco-2 cells and mice [62,63]. Deficient NPC1L1 in ApoE^−/−^ mice showed a robust decrease in cholesterol absorption and protection from the progression of atherosclerosis [64]. A human population-based study demonstrated that nonsynonymous variants in *NPC1L1* are associated with decreased cholesterol absorption and lower plasma LDL-C levels [41]. A multi-ancestry cohort study suggested that loss-of-function variants in *NPC1L1* are associated with reduced plasma LDL-C levels and a decreased risk of coronary heart disease [65]. These findings suggest that inhibiting NPC1L1 activity or expression reduces absorption of dietary cholesterol, thereby restricting enterocyte-mediated cholesterol metabolism and lipoprotein production.

Evidence from both animal studies and human genetic analyses has shown that hepatic NPC1L1 promotes the reabsorption of biliary cholesterol, leading to hepatic cholesterol accumulation. This accumulation is not only associated with hypercholesterolemia but has also been implicated in the development of hepatic steatosis. These findings highlight the critical role of NPC1L1 in regulating hepatic cholesterol metabolism and lipid deposition [11,66,67]. In animal models, transgenic mice expressing human NPC1L1 in hepatocytes (L1-Tg mice) show a 10~20-fold reduction in biliary cholesterol and a 30–60% increase in plasma cholesterol. Treatment with the NPC1L1 inhibitor ezetimibe normalizes these levels, indicating that dual inhibition of intestinal and hepatic NPC1L1 reduces plasma cholesterol and prevents excessive hepatic reabsorption of biliary cholesterol [68]. Comparative studies between *NPC1L1* knockout and wild-type mice have shown that deletion of *NPC1L1* attenuates high-fat diet (HFD)-induced hepatic lipid accumulation, suppresses genes involved in fatty acid biosynthesis and lipogenesis, and enhances insulin sensitivity. These findings suggest that reduced hepatic NPC1L1 expression improves hepatic lipid accumulation and ameliorates fatty liver [69]. In L1-Tg/LDLR^−/−^ mice, a HCD induced hyperlipidemia, where NPC1L1 overexpression reduced biliary cholesterol, increased plasma cholesterol and TG, and enhanced hepatic VLDL secretion into the circulation [70]. In L1-Tg mice, a two-week HFD markedly induced fatty liver. Along with increased biliary cholesterol reabsorption, hepatic retention of VLDL-TG was observed, leading to excessive lipid deposition in hepatocytes. These findings support a role for NPC1L1 in hepatic lipid accumulation and the pathogenesis of MAFLD [71,72].

In LDLR mutant (LDLR^mt^) mice fed a Western diet, those expressing NPC1L1 in hepatocytes developed more severe dyslipidemia and atherosclerotic plaque formation than mice expressing NPC1L1 only in enterocytes. This suggests that hepatic NPC1L1, together with intestinal NPC1L1, enhances cholesterol absorption, thereby exacerbating dyslipidemia and atherosclerosis [73]. In HFD-fed L1-Tg mice, hepatic NPC1L1-mediated cholesterol uptake promotes the formation of oxysterols, such as 22(R)-hydroxycholesterol and 25-hydroxycholesterol, which activate LXRα, driving lipogenesis and hepatic lipid accumulation and contributing to fatty liver development [74]. In Zucker obese fatty (ZOF) rats, inhibition or downregulation of NPC1L1 improves insulin sensitivity, suppresses hepatic gluconeogenesis, and reduces ROS, ER stress, and hepatic lipid accumulation. These findings suggest that NPC1L1 inhibition alleviates hepatic cholesterol accumulation and inflammation, offering therapeutic benefits for diabetes and fatty liver [75]. In L1LivOnly transgenic mice expressing human NPC1L1 exclusively in hepatocytes, elevated NPC1L1 suppresses biliary and fecal cholesterol excretion, increasing plasma cholesterol. Conversely, its inhibition enhances macrophage-mediated RCT and fecal cholesterol excretion, highlighting a key role for hepatic NPC1L1 in cholesterol elimination and in the treatment of hypercholesterolemia [76,77].

## 6. Natural Phytochemicals as NPC1L1 Inhibitors

**Figure 5:**
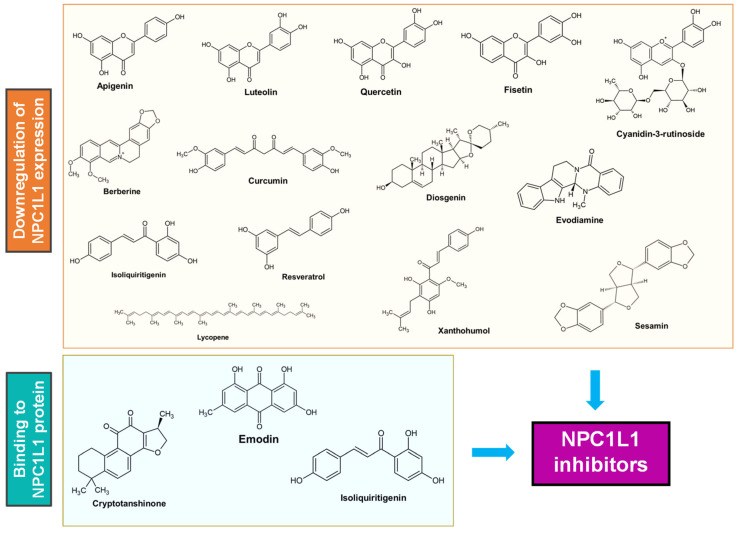
Mechanisms of action in the natural phytochemical-based NPC1L1 inhibitors. Natural phytochemical inhibitors of NPC1L1 primarily act by downregulating NPC1L1 gene expression, thereby reducing cholesterol uptake. Representative compounds include apigenin, berberine, curcumin, cyanidin-3-rutinoside, diosgenin, evodiamine, fisetin, isoliquiritigenin, luteolin, lycopene, quercetin, resveratrol, sesamin, and xanthohumol. In addition, certain compounds, such as cryptotanshinone, emodin, and isoliquiritigenin, can bind to NPC1L1 and inhibit its transport activity. Chemical structures were drawn using ACD/ChemSketch (Freeware) Version 2020.1.2 (Advanced Chemistry Development, Inc., Toronto, ON, Canada).

By targeting NPC1L1, cholesterol absorption inhibitors reduce intestinal cholesterol uptake and lower plasma and hepatic cholesterol levels. However, the only approved inhibitor, ezetimibe, demonstrates limited clinical efficacy in patients with hypercholesterolemia when used as monotherapy. Therefore, the development of novel agents that inhibit NPC1L1 expression or activity is essential to regulate intestinal and hepatic cholesterol uptake more effectively and to improve the prevention and treatment of ASCVD and MAFLD. Several phytochemicals have been reported to modulate cholesterol and lipid homeostasis and may be useful for the prevention and treatment of dyslipidemia [78,79]. Recently, phytochemicals with potential NPC1L1-inhibitory effects have been identified that lower cholesterol absorption by suppressing NPC1L1 transport and expression, representing an alternative strategy for modulating cholesterol metabolism [80]. Figure 5 and Table 1 summarize studies on phytochemicals that act as potential NPC1L1 inhibitors, highlighting their effects on the modulation of NPC1L1 expression or interaction of NPC1L1 to regulate transport activity in both in vitro and in vivo models.

### 6.1. Apigenin, Luteolin, and Quercetin

Apigenin, luteolin, and quercetin are dietary flavonoids with diverse health-promoting bioactivities [81]. In mice fed HFD supplemented with apigenin, hepatic levels of SREBP2 and HMGCR proteins are reduced. In contrast, luteolin supplementation decreases NPC1L1 expression while increasing ABCG5/8 expression in the intestinal mucosa. Together, these findings suggest that apigenin inhibits cholesterol biosynthesis, whereas luteolin promotes cholesterol elimination [82]. In hamsters fed a HCD, apigenin increases hepatic LDLR expression, reduces non-HDL cholesterol levels, and enhances fecal cholesterol excretion. It also suppresses NPC1L1 expression in the intestinal mucosa, thereby inhibiting cholesterol uptake and promoting its elimination [83]. Luteolin and quercetin reduce cholesterol absorption in Caco-2 cells and NPC1L1-transfected HEK293T cells by downregulating NPC1L1 mRNA expression. Consistently, cholesterol-lowering effects have also been observed in rats supplemented with luteolin or quercetin [84].

### 6.2. Berberine and Evodiamine

Berberine and evodiamine are bioactive compounds derived from the traditional medicinal plants *Coptidis rhizoma* and *Evodia rutaecarpa*, respectively [85,86]. Coadministration of these compounds to rats fed an HFD for four weeks significantly reduced serum total cholesterol, TG, LDL-C, and hepatic total cholesterol levels. Furthermore, this combination decreased intestinal cholesterol absorption by downregulating NPC1L1 expression in HFD-induced hyperlipidemic rats [87].

### 6.3. Cryptotanshinone

Cryptotanshinone is a lipophilic compound isolated from the dried root of *Salvia miltiorrhiza* Bunge (Danshen) [88]. In HFD-fed mice, cryptotanshinone significantly reduced hepatic steatosis and suppressed NPC1L1-mediated intestinal cholesterol absorption; however, it did not affect NPC1L1 mRNA levels. Furthermore, molecular modeling showed that cryptotanshinone interacts stably with NPC1L1 and modulates its molecular function. These findings suggest that cryptotanshinone acts as a direct NPC1L1 inhibitor, thereby ameliorating hepatic steatosis in MAFLD models [89].

### 6.4. Curcumin

Curcumin, the major bioactive polyphenol in turmeric, has been shown to regulate cholesterol homeostasis and possess anti-atherosclerotic properties [90]. It dose-dependently inhibits cholesterol uptake by downregulating NPC1L1 expression in intestinal Caco-2 cells [91]. In addition, curcumin suppresses NPC1L1 transcription by modulating SREBP2, further reducing cholesterol uptake in Caco-2 cells [92]. In animal studies, curcumin supplementation decreased the expression of SREBP-2 and NPC1L1, which may contribute to reduced serum cholesterol, TG, and LDL-C levels in hamsters fed an HFD [93]. Furthermore, curcumin attenuates HFD-induced fatty liver and hypercholesterolemia by downregulating NPC1L1 expression in both the intestine and liver through suppression of the SREBP-2/HNF1α pathway. This leads to reduced cholesterol absorption and biliary reabsorption, ultimately mitigating hepatic cholesterol accumulation and steatosis [94,95].

### 6.5. Cyanidin-3-Rutinoside

Cyanidin-3-rutinoside, a natural dietary anthocyanin abundant in blackberries, mulberries, and black raspberries, has been shown to regulate lipid metabolism. In Caco-2 cells, it significantly decreases cholesterol uptake from both free cholesterol and mixed micelles. Furthermore, cyanidin-3-rutinoside suppresses NPC1L1 mRNA expression, suggesting that it inhibits lipid absorption by downregulating NPC1L1 [96].

### 6.6. Diosgenin

Diosgenin, a plant-derived steroidal saponin structurally analogous to cholesterol, is a common precursor for steroid hormone synthesis and exhibits diverse pharmacological properties, including anti-inflammatory, lipid-lowering, and cholesterol-modulating effects [97]. In studies using Caco-2 cells and C57BL/6J mice fed a lithogenic diet, diosgenin supplementation inhibited intestinal cholesterol uptake by downregulating NPC1L1 expression. Mechanistically, diosgenin suppresses NPC1L1 transcription by directly inhibiting STAT3 phosphorylation. These findings suggest that diosgenin may help prevent cholesterol gallstone formation by reducing NPC1L1 expression and cholesterol absorption [98].

### 6.7. Emodin

Emodin, an anthraquinone phytochemical found in medicinal plants such as *Rheum rhabarbarum*, *Cassia*, and *Fallopia multiflora*, has demonstrated significant anti-atherogenic activity [99]. In HepG2 cells, emodin inhibits NPC1L1-mediated cholesterol uptake through an uncompetitive mechanism without altering NPC1L1 gene expression [100]. These findings suggest that emodin holds promise as a dietary supplement or lipid-modifying agent for the management of hypercholesterolemia.

### 6.8. Fisetin

Fisetin, a flavonoid abundant in strawberries, exhibits a broad spectrum of health-promoting activities, including potent anti-dyslipidemic effects [101]. In hypercholesterolemic mice, fisetin reduces serum total cholesterol, decreases hepatic lipid accumulation, and increases fecal neutral sterol content-findings that indicate enhanced transintestinal cholesterol excretion (TICE). Mechanistically, fisetin has been shown to activate the nuclear receptor PPARδ and downregulate NPC1L1 expression in Caco-2 cells [102]. Similarly, in ApoE^−/−^ mice fed a HFD, fisetin decreases NPC1L1 levels in the jejunum and stimulates TICE [103]. Collectively, these findings suggest that fisetin modulates cholesterol homeostasis by suppressing NPC1L1 expression and enhancing TICE, thereby alleviating hypercholesterolemia and atherosclerosis.

### 6.9. Isoliquiritigenin

Isoliquiritigenin, a chalcone flavonoid isolated from *Glycyrrhiza glabra*, has been shown to possess hypolipidemic and anti-atherogenic properties [104]. It downregulates NPC1L1 mRNA and protein expression in HepG2 cells and acts as a competitive inhibitor by binding to NPC1L1, thereby significantly inhibiting NPC1L1-mediated cholesterol uptake in HepG2 and Caco-2 cells [105].

### 6.10. Lycopene

Lycopene, a carotenoid abundant in tomatoes, plays a preventive role in cardiovascular disorders and supports overall health [106]. In Caco-2 cells, lycopene activates the nuclear receptor LXRα, which reduces NPC1L1 mRNA and protein levels in a dose-dependent manner, thereby inhibiting cholesterol absorption [107]. Furthermore, in ApoE^−/−^ mice fed an HFD, lycopene suppresses intestinal cholesterol absorption and attenuates atherosclerosis by downregulating HNF1α and NPC1L1 expression [108].

### 6.11. Resveratrol

Resveratrol, a natural polyphenolic compound abundant in grape skins, possesses well-documented cardioprotective effects [109]. In mice fed with HFD and HCD, resveratrol significantly downregulates intestinal NPC1L1 expression. In Caco-2 cells, LXRα knockdown has been shown to impair resveratrol-induced cholesterol excretion. These findings suggest that resveratrol reduces intestinal NPC1L1 expression by activating LXRα, thereby decreasing cholesterol absorption and lowering circulating cholesterol levels [110].

### 6.12. Sesamin

Sesamin is a natural cholesterol-lowering phytochemical abundant in sesame (*Sesamum indicum* L.) seeds [111]. In hamsters fed a sesamin-containing diet, the mRNA expression of intestinal *NPC1L1*, *ACAT2*, *MTTP*, and *ABCG5*/*ABCG8* was downregulated, accompanied by increased excretion of total neutral sterols. These findings indicate that sesamin modulates intestinal cholesterol absorption [112].

### 6.13. Xanthohumol

Several in vitro and in vivo studies have demonstrated that xanthohumol, a prenylated chalcone flavonoid primarily found in hops (*Humulus lupulus* L.), helps maintain cholesterol homeostasis and protects against hypercholesterolemia and atherosclerosis [113,114]. Xanthohumol significantly inhibits NPC1L1 mRNA and protein expression by downregulating HNF-4α, thereby reducing cholesterol absorption in Caco-2 cells. Additionally, it attenuates lovastatin-induced NPC1L1 overexpression in Caco-2 cells, suggesting its potential use in combination with statins as an effective therapeutic strategy for hypercholesterolemia [115].

Collectively, these findings indicate that phytochemicals possess the potential to inhibit NPC1L1 expression or activity, thereby modulating cholesterol homeostasis. By targeting this pathway, these compounds offer a promising therapeutic strategy for the management of hypercholesterolemia and MAFLD.

**Table 1:** The potential NPC1L1 inhibitors of natural phytochemicals.

Phytochemicals	Models	Mechanisms of Action	References
Apigenin	HCD-fed hamsters	Downregulation of NPC1L1 expression in intestine	[83]
Luteolin and quercetin	Caco-2 cells and Wistar rats	Downregulation of NPC1L1 expression	[84]
Berberine and evodiamine	HFD-fed Sprague-Dawley rats	Downregulation of NPC1L1 expression in intestine	[87]
Cryptotanshinone	HFD-fed mice Molecular docking	Inhibition of NPC1L1 Binding to NPC1L1 proteins	[89]
Curcumin	Caco-2 cells HFD-fed hamsters	Downregulation of NPC1L1 expression in intestine and liver	[91,92]
			[93,94,95]
Cyanidin-3-rutinoside	Caco-2 cells	Downregulation of NPC1L1 expression	[96]
Diosgenin	Caco-2 cells Lithogenic diet-fed mice	Downregulation of NPC1L1 expression in intestine	[98]
Emodin	HepG2 cells	Binding to NPC1L1 proteins/Uncompetitive inhibition of NPC1L1	[100]
Fisetin	Caco-2 cells HFD-fed ApoE^−/−^ mice	Downregulation of NPC1L1 expression in intestine	[102]
			[103]
Isoliquiritigenin	HepG2 cells and Caco-2 cells	Downregulation of NPC1L1 expression Binding to NPC1L1 proteins/Competitive inhibition of NPC1L1	[105]
Lycopene	Caco-2 cells HFD-fed ApoE^−/−^ mice	Downregulation of NPC1L1 expression in intestine	[107]
			[108]
Resveratrol	Caco-2 cells HFD- and HCD-fed mice	Downregulation of NPC1L1 expression in intestine	[110]
Sesamin	Hamsters	Downregulation of NPC1L1 expression in intestine	[112]
Xanthohumol	Caco-2 cells	Downregulation of NPC1L1 expression	[115]

## 7. Limitations and Future Perspectives

Although NPC1L1 is considered an important therapeutic target for hypercholesterolemia and metabolic disorders, and numerous phytochemicals have been reported to regulate NPC1L1 expression or activity, several limitations and physiological considerations should be taken into account when interpreting the current evidence.

Cholesterol is an essential component of cellular membranes. Consequently, excessive inhibition of NPC1L1-mediated cholesterol absorption may disrupt normal physiological functions and cholesterol homeostasis. In addition, long-term suppression of intestinal cholesterol uptake may trigger compensatory mechanisms, including increased endogenous cholesterol synthesis in the liver, underscoring the importance of balancing NPC1L1 activity when developing therapeutic agents targeting cholesterol metabolism.

Several currently reported phytochemicals may be more appropriately regarded as modulators of NPC1L1-associated cholesterol metabolism rather than fully validated direct NPC1L1 inhibitors. Among the phytochemicals discussed in this review, including terpenes, stilbenes, flavonoids, and their glycosides, reduced NPC1L1 expression and improvements in lipid metabolic abnormalities may reflect broader lipid metabolic reprogramming rather than direct inhibition of NPC1L1. Many phytochemicals exhibit pleiotropic lipid-regulatory effects by modulating transcriptional regulators, including SREBP2, LXRα, and PPARs, as well as lipid biosynthetic pathways and AMPK signaling. Current evidence remains insufficient to determine whether the observed effects on cholesterol homeostasis are directly mediated by NPC1L1 suppression or indirectly caused by alterations in other lipid metabolic pathways. Therefore, further mechanistic studies are needed to confirm that NPC1L1 is a direct molecular target of these phytochemicals to improve lipid metabolic disorders.

Additionally, current studies on phytochemicals for NPC1L1 inhibition provide only indirect evidence of NPC1L1 inhibition and do not clearly demonstrate a direct target to NPC1L1 proteins. To confirm whether phytochemicals directly bind to NPC1L1 and suppress its activity, additional studies using methods such as surface plasmon resonance (SPR), isothermal titration calorimetry (ITC), and high-resolution structural analyses, including cryo-EM, are needed.

Another important consideration involves the translational relevance of the experimental models used in current studies. Species-specific differences in hepatic NPC1L1 expression between humans and mice may affect the clinical translation of experimental findings. Notably, mouse livers lack NPC1L1, meaning that mouse models cannot fully recapitulate human hepatic cholesterol absorption and biliary cholesterol reuptake mechanisms. Therefore, findings derived from NPC1L1 studies using Caco-2, HepG2 cells, ApoE^−/−^ mice, high-fat/high-cholesterol diet-fed mice, rats, or hamster models may have limitations when translated to human cholesterol metabolism and the preliminary pharmacological evaluation of NPC1L1 inhibitors. Future studies incorporating humanized NPC1L1 animal models, primary human hepatocytes, or organoid systems, together with direct target-binding assays for validation, will be essential to strengthen mechanistic insights with conclusions relevant to translational medicine.

## 8. Conclusions

The molecular mechanisms underlying intestinal and hepatic NPC1L1 regulation have been elucidated, paving the way for the discovery of inhibitors that modulate cholesterol absorption and metabolism to treat hypercholesterolemia and hepatic steatosis, as well as for potential alternatives to conventional therapies. Studies on the cholesterol-modulating effects of phytochemicals, achieved by inhibiting NPC1L1 expression or activity, are relatively recent. This review highlights that phytochemicals capable of regulating NPC1L1 expression may serve as novel therapeutic agents, particularly for individuals who fail to achieve LDL-C targets or are intolerant to statins. The diversity of phytochemicals targeting NPC1L1 through distinct mechanisms presents valuable opportunities for precision medicine. These promising lipid-lowering effects of NPC1L1 inhibitors warrant further clinical investigation.

## Data Availability

No new data were created or analyzed in this study. Data sharing is not applicable to this article.
